# Higher everyday functioning, lower quality of life, and home care are associated with persisting symptoms after a COVID-19 infection in German care recipients

**DOI:** 10.3389/fpubh.2025.1559778

**Published:** 2025-05-15

**Authors:** Martin J. Koch, Peter K. Kurotschka, Dagmar Holmer, Christine Eidenschink, Tobias Dreischulte, Anita Hausen, Michael Hoelscher, Christian Janke, Thomas Kühlein, Armin Nassehi, Daniel Teupser, Jochen Gensichen, Ildikó Gágyor

**Affiliations:** ^1^Department of General Practice, University Hospital Würzburg, Würzburg, Germany; ^2^Institute of General Practice and Family Medicine, University Hospital LMU Munich, Munich, Germany; ^3^Faculty of Health and Nursing, Catholic University of Applied Sciences Munich, Munich, Germany; ^4^Division of Infectious Diseases and Tropical Medicine, University Hospital LMU Munich, Munich, Germany; ^5^Institute of General Practice, Friedrich-Alexander-University of Erlangen-Nuremberg, Erlangen, Germany; ^6^Institute of Sociology, LMU Munich, Munich, Germany; ^7^Institute of Laboratory Medicine, University Hospital LMU Munich, Munich, Germany

**Keywords:** persisting symptoms, COVID-19, care recipients, everyday functioning, quality of life, home care

## Abstract

**Introduction:**

To date, risk factors for persisting symptoms after a COVID-19 infection have not been investigated in people needing care or support. Prior meta-analyses identified age, obesity, and female sex as risk factors for persisting symptoms after a COVID-19 infection in the general population.

**Methods:**

This study is part of the Bavarian ambulatory COVID-19 monitor. Data were collected from ambulatory patients needing care/support and a past COVID-19 infection. Different exposure measures (age, sex, body-mass index, income, packyears, smoker status, relationship status, type of care, care level, educational and vocational qualification, quality of life, health status, functioning, depression, cognitive abilities, anxiety) and persisting symptoms after COVID-19 (≥ 1 symptom with a duration of ≥12 weeks following a COVID-19 infection) were collected. Bivariate analyses and multiple logistic regression with multiple imputations were used to investigate the association between exposure and persisting symptoms.

**Results:**

We included 514 participants (COVID-19 infection, needing care/support, completed questions on persistent symptoms). 68.3% were female, with a mean age of 80.5 years (range: 24–103 years). The sample is characterized by the need for support (i.e., degree of impairment of independence or frailty score ≥ 5). Bivariate analyses revealed associations of everyday functioning, depression, cognitive functioning, living in a relationship, care level, educational qualification, vocational qualification, and type of care with persisting symptoms. In multiple logistic regression, a higher level of functioning (OR = 2.72, 95%-CI: 1.20, 6.17), the quality of life (OR = 1.12, 95%-CI: 1.03, 1.23), and the type of care (OR = 3.16, 95%-CI: 1.48, 6.73) were significantly associated with persisting symptoms.

**Discussion:**

This is one of the first studies investigating the risk factors for persisting symptoms after COVID-19 in people in need of care or support. The risk factors in our study (everyday functioning, depression, cognitive functioning, living in a relationship, care level, educational qualification, vocational qualification, and type of care) differ from those identified in prior meta-analyses on the general population (age, obesity, female sex; these were not significant in our study). Our study highlights the importance of considering vulnerable groups in particular from the outset of future pandemic or epidemic events.

## 1 Introduction

During the COVID-19 pandemic and until today, there were more than 770 million reported infections and 7 million reported deaths worldwide (1). Even after the pandemic phase has subsided and the number of acute infections has decreased compared to the previous years, many patients report long-lasting symptoms. If symptoms persist for longer than 12 weeks after the beginning of a COVID-19 infection, post-COVID-19 syndrome can be the cause (2, 3). The prevalence of post-COVID-19 syndrome in adult COVID-19 survivors is estimated to be around 42 and 51% in two meta-analyses (4, 5). Among the most important symptoms are fatigue, cognitive impairments (e.g., problems concentrating), cough, shortness of breath, altered taste, pain, anxiety, depressive symptoms, and sleep problems (6, 7). This might heavily impact the quality of life of those affected. Simultaneously, diagnosis of post-COVID-19 is challenging. Attribution of symptoms to previous COVID-19 infection remains clinically challenging (8). Individuals with care needs are underrepresented in prior research, especially in meta-analyses on the risk factors related with persisting symptoms.

Several meta-analyses are concerned with risk factors of post-COVID-19 symptoms. A first meta-analysis [studies published until mid-2022 (9),] showed that female sex and hospitalization were associated with a higher risk of post-COVID-19 in general population studies. The duration of the hospitalization was also associated (participants with persistent symptoms stayed in hospitals significantly longer). Chronic obstructive pulmonary disease and the number of comorbidities were not associated with a higher risk of post-COVID-19 symptoms. Notarte et al. conducted a meta-analysis on age and sex as risk factors for post-COVID-19 in 2022 (10). They did not find a significant effect of age, neither when comparing groups lower/older than 60 years or when comparing average age values. They, however, found that female sex was significantly associated with a higher risk of post-COVID-19. In a more comprehensive meta-analysis (11), Tsampasian et al. included several potential risk factors from studies until the end of 2022. Female sex, obesity (BMI ≥ 30), and smoking were associated with a higher risk of post-COVID-19 symptoms. Older participants in two groups (group 1: 40–69 and group 2: > 70) both showed higher risks compared to participants younger than 40 years. Concerning comorbidities, individuals with anxiety and/or depression, asthma, chronic obstructive pulmonary disease, diabetes, immunosuppression, and ischemic heart disease showed a higher risk of post-COVID-19 symptoms (only chronic kidney disease was not significantly associated with a higher risk). Participants who were hospitalized during their COVID-19 infection and participants who were in the intensive care unit showed higher risks compared to individuals who were not. Participants who were vaccinated (2 doses) had a significantly lower risk of developing post-COVID-19 symptoms.

Based on these meta-analyses, for the current study, we expect that female sex, obesity (BMI ≥ 30), smoking, and anxiety/depression are relevant factors associated with persisting symptoms after a COVID-19 infection. However, none of the identified meta-analyses explicitly includes people with care needs. We also did not find reviews or single empirical studies on this topic. While several studies on risk factors of persisting symptoms after a COVID-19 infection in the general population of all ages exist, we have little knowledge about these factors in individuals in need of care. To date, persisting symptoms have rarely been investigated in the older adult and people in need of care (12). Our aim was to identify factors associated with risk factors for persisting symptoms after a COVID-19 infection in this population.

## 2 Materials and methods

### 2.1 Study

This study’s cross-sectional data were collected as part of the Bavarian outpatient COVID-19 Monitor (BaCoM) (13) between March 2021 and December 2023. Baseline and follow-up data were collected on 1,000 participants across three study sites in Bavaria (Munich, Erlangen, and Würzburg). The aim was to record the physical and psychosocial impact of the COVID-19 pandemic on people in need of care or support. Comprehensive baseline data collection was conducted for all participants, including a thorough physical examination and the use of standardized questionnaires. In addition, follow-up examinations were carried out every 6 months to record the development of the parameters examined over time. For participants who left the trial, the reason and period of the survey were documented. Only baseline data were needed for the current study. We only used baseline data because it included all necessary information on exposure and outcome variable and was more complete compared to the follow-up data. The baseline data were collected by the trial team within 4 weeks of recruitment by using questionnaires on site. The Bavarian State Ministry of Health, Care, and Prevention funded the project.

### 2.2 Sample

**Figure 1:**
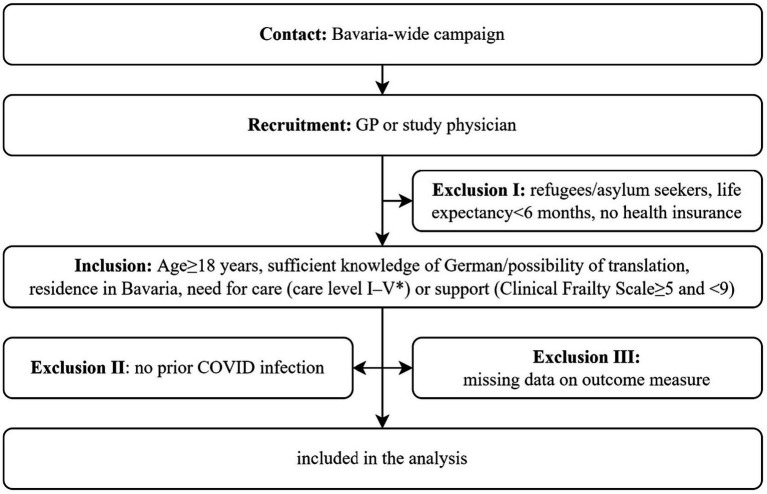
Flow diagram of included participants in BaCoM.

For this analysis, we included people in need of care or support with a positive SARS-CoV-2 PCR test (maximum backdated to 1 March 2020) from the Bavarian ambulatory COVID-19 monitor (13) who answered the questions on persistent symptoms after the infection. Participants were identified in nursing homes or outpatient care settings via a Bavaria-wide recruitment campaign. We contacted potential participants with cold calling via letterbox distribution and telephone, GP waiting rooms, and pharmacies. Irrespective of how prospective participants were identified, they were recruited and consented by their GP or a study physician, i.e., a member of the study team, from the involved academic departments. Included were individuals aged ≥ 18 with sufficient knowledge of German or possibility of translation. Further, a residence in Bavaria and a need for care (care level I-V) or support (clinical frailty score of 5 to 9) were required. Care level I-V includes individuals with a low to severe impairment of independence in the German healthcare system. A clinical frailty score of 5 to 9 includes individuals who are at least obviously slowed down in their activities and need help with demanding everyday activities. At the highest level, these individuals are terminally ill. Refugees/asylum seekers were excluded due to insufficient knowledge of German and because participation was not certain until the end of the study due to possible repatriation. Individuals without a health insurance were also excluded. These limits were implemented to protect scarce resources during the pandemic. For the current analysis, individuals without a prior COVID infection or missing data on the outcome measure were excluded. Figure 1 illustrates the flow of included participants described above with inclusion and exclusion criteria to enter the study cohort.

### 2.3 Measures

#### 2.3.1 Exposure

Sociodemographic information of all participants was recorded (incl. age, sex, income, body mass index [BMI], relationship status [yes/no]). They were asked to report on their educational (basic secondary school, secondary school, A-levels) and vocational qualification (no qualification, vocational school/apprenticeship, specialist/technician/master school, engineering school, university/university of applied science). We further collected information on health-related behavior (packyears [number of daily packs smoked multiplied by the number of years], smoker status [yes/former/no]). The participants were asked to report the type of care (nursing home, home care, outpatient care) and care level (a German system categorizing the level of independence: I: minor impairment of independence, II: significant impairment of independence, III: severe impairment of independence, IV: most severe impairment of independence, V: most severe impairment of independence with special requirements for nursing care).

Quality of Life was measured with the European Quality of Life 5 Dimension 5 Level [EQ-5D-5L; (14)] scale. This patient-reported score measures the general quality of life of patients independently of specific diseases or medical conditions across five dimensions. Each dimension is rated on a scale of 1 to 5. The scales are labeled according to the specific dimension. We calculated a sum score to represent the participants’ general quality of life in one numerical score. Higher values indicate a lower quality of life.

Self-assessed health was assessed with the visual assessment scale (EQVAS) of the EQ-5D-5L. Participants assess their own health on a visual scale of 0 to 100.

Everyday functioning was measured using the Barthel index (15). Research nurses from the involved academic departments assessed 10 different areas of everyday functioning. Participants receive 0 or 5 points for the areas of bathing and grooming, 0, 5, or 10 points for feeding, dressing, bowel control, bladder control, toilet use, and stairs, and 0, 5, 10, or 15 points per item for transfers and mobility. A sum score was calculated. A total score of 100 points is possible, higher scores show a higher level of everyday functioning.

The Patient Health Questionnaire-9 [PHQ-9, German version, (16)] was utilized to measure symptoms of depression in the participants. The scale consists of nine items assessing symptoms of depression. Participants rated how frequently they experienced these symptoms in the last 2 weeks on a scale from 0 to 5 (“Not at all,” “On some days,” “On more than half of the days,” “Almost every day”). We calculated a sum core for each participant.

The Six Item Screener [SIS, (17, 18)] is a short instrument to measure cognitive abilities. Participants were asked to report the current year, month, and day of the week. They are further asked to remember three words for a short period of time. A correct task is scored with one point; a sum score is calculated. A total of six points is possible.

The Montreal Cognitive Assessment Blind [MoCA, (19)] was also used to measure cognitive abilities if the SIS score was ≥ 4. This approach should reduce the amount of work involved in the survey. In order to obtain a complete picture of cognitive health, SIS and MoCA values were used for the current study. The MoCA consists of 30 different tasks. We included 22 tasks that were asked via telephone without the need for visual material. Normally, a total score is calculated with a possible maximum score of 30 points. Here, 22 points were possible.

We assessed possible symptoms of a generalized anxiety disorder utilizing the German version of the Generalised Anxiety Disorder Scale 7 [GAD-7; (20)]. Participants rated how frequently they experienced these symptoms in the last 2 weeks on a scale from 0 to 5 (“Not at all,” “On some days,” “On more than half of the days,” “Almost every day”). We calculated a sum core for each participant.

We dichotomized most metric variables (BMI, Barthel index, PHQ-9, SIS, MOCA, GAD-7) following cut-off scores defined in the literature in order to better represent the clinical relevance of the construct captured (all cut-offs can be seen in Table 1). If all participants were above or below a cut-off, we performed a median split instead. This was the case for the Barthel index and the MOCA. No cut-off scores were available for the EQ5D5L and the EQVAS scores; we did not dichotomize both scores. All instruments have been used with older patients/individuals in need of care before while some of them were explicitly developed to assess health in this population (e.g., SIS, MoCA).

**Table 1:** Overview of the exposure variables and association with the occurrence of at least one persisting symptom following a COVID-19 infection.

Variable		No persisting symptoms			persisting symptom(s)			*p*	*t*
	*n*	*M*	*SD*	*n*	*M*	*SD*			
Age (years)		398	80.78	12.93	114	79.46	10.92	0.322	0.99
Income (in 100€)		230	17.59	11.93	74	17.94	11.05	0.823	−0.22
Packyears		191	16.93	25.18	61	16.66	26.27	0.942	0.07
Quality of life (EQ-5D-5L)		393	11.70	4.54	113	12.14	4.54	0.363	−0.99
Self-assessed health (EQVAS)		374	60.77	21.46	113	57.45	20.68	0.147	1.45
		*n*	%		*n*	%		*p*	ρ
BMI	< 30	284	76.55		79	69.30		0.119	0.07
	≥ 30	87	23.45		35	30.70			
Functioning (Barthel index)	< 85	202	51.79		34	30.36		**< 0.001**	**0.18**
	≥ 85	188	48.21		78	69.64			
Depression (PHQ-9)	< 10	316	83.38		82	74.55		**0.036**	**0.10**
	≥ 10	63	16.62		28	25.45			
Cognitive ability (SIS)	< 4	57	15.36		4	3.51		**< 0.001**	**0.15**
	≥ 4	314	84.64		110	96.49			
Cognitive ability (MoCA)	< 18	146	45.91		32	29.36		**0.002**	**0.15**
	18	172	54.09		77	70.64			
Anxiety (GAD)	< 10	336	91.06		99	89.19		0.554	0.03
	≥ 10	33	8.94		12	10.81			
In a relationship	No	295	74.12		62	54.87		**< 0.001**	**0.17**
	Yes	103	25.88		51	45.13			
Sex	Female	277	69.25		74	64.91		0.380	0.04
	Male	123	30.75		40	35.09			
		*n*	%		*n*	%		*p*	ρ
Care level	I	27	9.61		14	23.33		**0.003**	**−0.15**	II	89	31.67		20	33.33				III	108	38.43		21	35.00				IV	42	14.95		4	6.67				V	15	5.34		1	1.67			
	Educational Qualification	No Qualification	13	3.35		1	0.88		**0.027**	**0.09**
		Basic secondary school	198	51.03		52	46.02			
		Secondary school	109	28.09		27	23.89			
		A-levels	68	17.53		33	29.20			
Vocational qualification	No qualification	90	23.50		23	21.10		**0.020**	**0.10**
	Vocational school/apprenticeship	202	52.74		45	41.28			
	Specialist/technician/master school	39	10.18		14	12.84			
	Engineering school	8	2.09		2	1.83			
	HAW/university	44	11.49		25	22.94			
		*n*	%		*n*	%		*p*	ρ
Type of care	Nursing home	218	61.76		24	26.37		**<0.001**	**0.29**	Home care	112	31.73		57	62.64				Outpatient care	23	6.52		10	10.99			
	Smoker	Yes	30	7.85		4	3.54		0.108	0.10	Former	117	30.63		44	38.94				No	235	61.52		65	57.52			

Care level: I: minor impairment of independence, II: significant impairment of independence, III: severe impairment of independence, IV: most severe impairment of independence, V: most severe impairment of independence with special requirements for nursing care. Bold values indicate a significant association.

#### 2.3.2 Outcome

Individuals in need of care with a positive SARS-CoV-2 PCR test (maximum backdated to 1 March 2020), were asked to report the symptoms they have or have had after their COVID-19 infection. They chose their symptoms from a list of the following 31 symptoms: fatigue and exhaustion, cough, shortness of breath, odor disturbances, loss of taste, headache, muscle pain, joint pain, chest pain, cognitive impairment, fever, sore throat, runny nose, stuffy nose, dizziness, nausea, diarrhea, abdominal pain, loss of appetite, skin changes, swelling of lymph nodes, vomiting, sneezing, earache, wheezing, nerve pain, visual disturbances, ataxic gait, disorientation and confusion, speech disorders, eye movement disorders. They were able to report further symptoms, which were, however, not considered for the current analysis. They further reported the duration of weeks of each of the reported symptoms. The self-report on symptoms and symptom duration was not corroborated by clinical documentation. We then checked if a participant reported at least one symptom with a minimum duration of 12 weeks. If they did, they were assigned to the group of individuals with persisting symptoms after a COVID-19 infection (primary outcome measure).

### 2.4 Analysis

We report mean values and standard deviations for all metric and absolute frequencies for categorical variables. We test for bivariate relationships between the exposure variables and the occurrence of persisting symptoms after a COVID-19 infection (at least one persisting symptom, duration ≥ 12 weeks) with different analyses. For metric exposure variables (age, income, packyears, quality of life, self-assessed health), we implemented t-tests for independent samples to test for group differences between participants with and without persisting symptoms. We calculated *ρ*-correlations for dichotomous categorical variables and bi-serial rank correlations (τ_b_) for ordinal variables. For categorical variables, we calculated Cramér’s V.

Because of the high number of missing values (10.06%), we implemented multiple imputations to estimate the missing values before the multivariate analysis. Multiple imputation with fully conditional specification [Markov Chain Monte Carlo method (21, 22)] in IBM SPSS created 10 complete data sets. We assumed that compared to 100 imputations, a power falloff of <3% might be acceptable. This would require only 5 imputations (23). By using 10 imputation samples, we ensured that power falloff should not be an issue for our analysis. We followed some specifications for the imputation: Metric variables that were to be dichotomized were first imputed and then dichotomized. To ensure that all values fall within reasonable boundaries, we set constraints for the estimation of the values for which explicit boundaries exist (e.g., due to possible maximum scores of questionnaires). We calculated BMI after the imputation from the values for weight and height.

To adjust for confounding, including the 10 imputed data sets and the original data set, we tested the multivariable relationship between the exposure variables and the occurrence of persisting symptoms after a COVID-19 infection by calculating a multiple logistic regression. In multiple imputation, a separate regression analysis was run for each data set. Then, the findings are aggregated. We included all exposure variables as described above as independent variables in the regression analysis. The metric and dichotomized variables were directly included in the analysis. The categorical variables were dummy-coded and then included in the analysis (reference categories are shown in Table 2, marked in italics). The overall model was evaluated considering a Chi^2^-test, Nagelkerkes R^2^, and area under the receiver operating characteristic curve (AUC) based on the predicted probabilities. Additionally, odds ratios (OR), a *p*-value, 95% confidence intervals (CI) for ORs were reported for each independent variable. For all analyses, we used a significance level of α < 0.05. In an exploratory manner, we tested for interactions between all exposure variables and gender, age, and level of care.

**Table 2:** Effects of the logistic regression for the occurrence of at least one persisting symptom following a COVID-19 infection.

Exposure measures		95%-CI		
	OR	lo	up	*p*
Age (years)	1.00	0.98	1.03	0.814
Sex (female = 0, male = 1)	1.11	0.56	2.21	0.762
BMI	1.45	0.83	2.52	0.191
Income	1.00	1.00	1.00	0.373
Packyears	1.00	0.98	1.02	0.863
Functioning (Barthel index)	**2.72**	**1.20**	**6.17**	**0.017**
Depression (PHQ)	1.47	0.74	2.94	0.275
Cognitive ability (SIS)	2.87	0.86	9.50	0.085
Cognitive ability (MOCA)	1.61	0.87	3.00	0.131
Anxiety (GAD)	0.56	0.22	1.45	0.233
Quality of life (EQ-5D-5L)	**1.12**	**1.03**	**1.23**	**0.013**
Self-assessed health (EQVAS)	1.00	0.99	1.01	0.893
In a relationship (no = 0, yes = 1)	1.07	0.57	2.01	0.824
Care level I vs.				
Care level II	0.63	0.25	1.62	0.332
Care level III	0.66	0.22	2.00	0.459
Care level IV	0.32	0.07	1.44	0.135
Care level V	0.77	0.09	6.60	0.806
Smoker vs.				
Former smoker	2.45	0.68	8.87	0.173
Non-smoker	2.01	0.51	8.00	0.319
Basic secondary school vs.				
No qualification	0.16	0.01	2.30	0.176
Secondary school	0.59	0.28	1.21	0.148
A-levels	0.72	0.25	2.09	0.547
Vocational school/apprenticeship vs.				
No qualification	1.49	0.74	3.02	0.264
Specialist/technician/master school	1.19	0.49	2.93	0.700
Engineering school	1.92	0.33	11.28	0.468
(applied) university	2.41	0.81	7.18	0.115
Nursing home vs.				
Home care	**3.16**	**1.48**	**6.73**	**0.003**
Outpatient care	2.69	0.98	7.37	0.054

OR = Odds Ratio, 95%-CI = 95% Confidence interval of the OR, Cut-Offs: BMI: <30 vs. ≥30, functioning (Barthel index): <85 vs. ≥85, depression (PHQ): <10 vs. ≥10, cognitive ability (SIS): <4 vs. ≥4, cognitive ability (MOCA): <18 vs. ≥18, anxiety (GAD): <10 vs. ≥10. Bold values indicate a significant association.

## 3 Results

### 3.1 Participants

The sample consisted of N = 514 participants from Germany. The mean age of participants was 80.5 years (SD = 12.5). The oldest participant was 103 and the youngest was 24 years old (range = 79). More than two-thirds of the participants were female (68.3%). The average self-reported health was 60.0 out of 100 points (SD = 21.3). 25.2% of all participants had a BMI ≥ 30. 114 participants (22.8%) reported at least one persisting symptom (duration ≥ 12 weeks). 54.5% of patients were in a nursing home, 31.1% in home care, and only 7.4% in outpatient care. Most patients were non-smokers (60.6%) or former smokers (32.5%). Only 6.9% were actively smoking. An overview of all exposure variables for the groups with and without persisting symptoms can be seen in Table 1.

### 3.2 Bivariate analyses

In the bivariate analyses (Table 1), higher scores of everyday functioning (*p* < 0.001), higher scores of depression (*p* = 0.036), a higher cognitive functioning (SIS: *p* < 0.001 and MoCA: *p* = 0.002), and living in a relationship (*p* < 0.001) were positively associated with the occurrence of persisting symptoms (all cut-offs can be seen in Table 1). The care level was negatively associated with persisting symptoms after a COVID-19 infection (*p* = 0.003). Educational and vocational qualifications were both positively correlated with persisting symptoms (*p* = 0.027 resp. = 0.020). Age, income, packyears, quality of life, self-assessed health, BMI, anxiety, sex, and smoking were not significantly associated with persisting symptoms.

### 3.3 Multivariate analysis

The multiple logistic regression yielded a significant result (average across all imputations χ^2^(28) = 102.2, all *p* < 0.001). A significant proportion of variance was explained (average across all original and imputed data sets: Nagelkerkes *R^2^* = 28.2%). Multicollinearity was not a problem, as all variables showed tolerance values > 0.1 in all data sets. The mean AUC was 0.81 across all imputed datasets (range: 0.79–1.00). A higher level of functioning, quality of life, and type of care were significantly associated with the occurrence of persisting symptoms after a COVID-19 infection (Table 2). A level of functioning above or equal to the median value (i.e., ≥ 85 points on the Barthel index) was associated with an increased risk of persisting symptoms by a factor of 2.7 If the quality of life is reduced by one point (i.e., the scale increases by one point), the chance of persisting symptoms is increased by the factor 1.1. In addition, in home, the chance of having persisting symptoms was 3.2 times higher compared to participants in nursing homes. Age, income, packyears, depression, BMI, anxiety, sex, smoking, cognitive ability, relationship status, care level, school education, and vocational education were not associated with a higher risk of persisting symptoms. We found no significant interaction terms between gender, age, the level of care, and the other exposure measures.

## 4 Discussion

### 4.1 Summary of main findings

In bivariate analyses, all effects were small (i.e., effect sizes < 0.30). The type of care showed the strongest association with persisting symptoms following a COVID infection. Positive correlations, in descending order of strength, included higher everyday functioning, being in a relationship, higher cognitive ability, depression, educational qualification and vocational qualifications, female sex, and anxiety. Care level (degree of independence impairment) was the only variable negatively associated with persisting symptoms. Multivariable analysis identified three factors significantly associated with persisting symptoms: higher functioning, lower quality of life, and type of care. The largest effect was found for the type of care with a more than threefold higher risk of persisting symptoms for individuals in home care compared to individuals in a nursing home.

### 4.2 Comparison with existing literature

Based on meta-analyses on risk factors of persisting symptoms after a COVID infection, we expect that female sex, age, obesity (BMI ≥ 30), smoking, and depression are relevant factors associated with persisting symptoms after a COVID-19 infection. While female sex was identified as a risk factor in all meta-analyses (9–11), it was no risk factor for the care recipients of our study. One meta-analysis showed that age is a risk factor (11), while in another meta-analysis (10) and in our study it was not. This might possibly be due to different cut-offs and age groups that are compared in the meta-analyses and our study. We included age as a metric variable and calculated the mean difference (or the effect of the metric variable in the logistic regression) in a rather old group of care recipients. Age differences might only become significant when considering a larger age range or when comparing specific categorical age groups (11). Obesity (BMI ≥ 30) was a risk factor in the same meta-analysis (11) but not in our study. Smoking was not associated with persisting symptoms in our study, neither when operationalized as a categorical (yes/former/no) or metric (packyears) variable. This is contrary to one meta-analysis that identified smoking as a risk factor (11). The same meta-analysis also identified anxiety/depression as a risk factor. This finding could not be confirmed in our study.

Some of the differences between our study’s results and the findings of the meta-analyses are likely due to differences between our specific target population (i.e., individuals in need of care/support) and the general population. The meta-analyses focus on the general population which might also include individuals in need of care/support. Our study, however, was explicitly targeted at only included individuals of this population. It seems not unsurprising that the different methods (meta-analysis and a single population study) come to different results. Further, the meta-analyses and our study focus on different times of the COVID pandemic. The meta-analyses only included studies that were published in or before 2022 indicating that the data were collected even before this point in time. Our study’s data were collected between March 2021 and December 2023. There is therefore a partial overlap in the time periods of the meta-analyses and our study. However, the meta-analyses also cover studies from earlier periods up to the beginning of 2020, while our study is also based on data from 2023. Different COVID variants dominated during these periods (24). This difference in time might, thus, also explain some of the differences in the identified risk factors.

### 4.3 Additional associations

In addition to the potential risk factors identified in the meta-analyses, we considered further variables as potential risk factors. It is important to note again, however, that the following considerations are not based on literature. Some of these are specific to individuals in need of care/support such as the type of care or are more relevant for this population such as everyday functioning. Everyday functioning (measured with the Barthel Index) can be seen as an indicator of independence in lifestyle and participation. Prima facie, it seems counterintuitive that individuals with higher everyday functioning have an increased risk of persisting symptoms because these individuals can be considered healthier. It is possible that people with high everyday functioning were more active during an infection and thus increased their risk of persistent symptoms. Alternatively, it seems conceivable that patients with higher everyday functioning are more likely to report new persisting symptoms following a COVID infection compared with individuals who have lower functioning and, thus, possibly lower health and more symptoms even before a COVID infection. More surprisingly, individuals who were cared for at home showed a higher risk compared with individuals who lived in a nursing home. Again, it seems possible that healthier people (i.e., people living at home) are more active or more likely to notice newly developed symptoms. Quality of life, however, is not specific to people in need of care/support. The association of quality of life and the occurrence of persisting symptoms after a COVID infections comes as no surprise. It seems reasonable to assume that individuals who suffer from persisting symptoms also show an impacted quality of life. A causal relationship between these factors seems possible.

### 4.4 Strengths and limitations

This is one of the first studies that investigated risk factors of persisting symptoms after a COVID-19 infection in a group of people in need of care or support. Further, we followed the WHO’s definition and other international studies when operationalizing the outcome of the current study. We used a multivariate logistic regression (in addition to bivariate analyses), which explained a fair amount of variance. We had a low level of missing data and implemented multiple imputations with established methods.

As our study is based on cross-sectional data, the independent associations found might not be indicative of causal or predictive effects. Residual confounding or reverse effects are possible. Longitudinal data (i.e., including the follow-up) may be more amenable to interpretation as causal or predictive effects. The data collection was difficult due to the high age of the participants, as some had difficulties recalling the required information and were burdened by the scope of the data collection. The high age might make it difficult to distinguish whether symptoms already existed before infection or whether they only appeared afterward (8) in an older sample of people in need of care. Faulty or inaccurate memories and reconstructions of time sequences might lead to erroneous data on the exact onset of a symptom after an infection (recall bias). In general, it seems questionable which symptoms are associated with COVID, as this is an old cohort. The long survey period was accompanied by different types of viruses, changing availability of care services (e.g., tests, masks, and vaccinations), changes in political and societal rules of interaction with patients, and other context factors. This makes it difficult to standardize and evaluate the findings. Also, the questionnaire took about 2 h to complete making it difficult for older individuals to concentrate. Further, prospective observation was not possible, as many people were not reached, and data collection was problematic during the process. Refugees/asylum seekers and people without health insurance were excluded due to scarce resources, which somewhat limited the scope of the study. The number of participants recruited via different recruitment channels were not documented.

### 4.5 Conclusion and further research

This is one of the first studies on factors associated with the occurrence of persisting symptoms after a COVID-19 infection in individuals in need of care or support. It is possible that this population also suffered physical consequences from the pandemic. If so, other factors were predictive of this compared to the normal population. During the COVID-19 pandemic, research on risk factors has been conducted; however, individuals requiring care or support have been largely underrepresented. To better address the challenges of future pandemics or epidemics, it is crucial to prioritize the inclusion of vulnerable groups from the outset.

## Data Availability

The raw data supporting the conclusions of this article will be made available by the authors, without undue reservation.

## Group members of the BaCoM-Study Group

Principal Investigator and Co-Principal Investigators: Jochen Gensichen* (PI), Tobias Dreischulte*, Ildikó Gágyor*, Anita Hausen, Michael Hoelscher, Christian Janke, Thomas Kühlein*, Armin Nassehi, Daniel Teupser.

Florian Arend, Felix Bader, Barbara Daubner, Christine Eidenschink, Caroline Floto, Dagmar Hindenburg, Sylvi Hoffmann, Peter Kurotschka, Daniela Lindemann, Karoline Lukaschek, Katharina Mayr, Irina Michel, Susan Müller, Marietta Rottenkolber, Linda Sanftenberg, Jessica Scheel-Barteit, Rita Schwaiger, Maria Sebastia͂o, Sabrina Weigand, Domenika Wildgruber, Susanne Winter, Isabel Zöllinger, Heidi Hentschel, Christina Huber, Julian Mayrhuber, Mara Pettke, Sophia Straub, Alexander Theiss.

* = study coordinator in recruiting centers.

## References

[1] World Health Organization. (2024). WHO COVID-19 dashboard. Available online at: https://covid19.who.int (Accessed October 1, 2024).

[2] Soriano JB Murthy S Marshall JC Relan P Diaz JV. A clinical case definition of post-COVID-19 condition by a Delphi consensus. Lancet Infect Dis. (2022) 22:e102–7. doi: 10.1016/S1473-3099(21)00703-9, PMID: 34951953 PMC8691845

[3] NICE. (2024). COVID-19 rapid guideline: managing the long-term effects of COVID-19. Available online at: https://www.nice.org.uk/guidance/ng188/resources/covid19-rapid-guideline-managing-the-longterm-effects-of-covid19-pdf-66142028400325 (Accessed October 1, 2024). 33555768

[4] ECDC. (2022). Prevalence of post COVID-19 condition symptoms: a systematic review and meta-analysis of cohort study data, stratified by recruitment setting.

[5] Sk Abd Razak R Ismail A Abdul Aziz AF Suddin LS Azzeri A Sha’ari NI. Post-COVID syndrome prevalence: a systematic review and meta-analysis. BMC Public Health. (2024) 24:1785. doi: 10.1186/s12889-024-19264-5 38965510 PMC11223303

[6] Fernandez-de-las-Peñas C Notarte KI Macasaet R Velasco JV Catahay JA Ver AT. U. A. Persistence of post-COVID symptoms in the general population two years after SARS-CoV-2 infection: a systematic review and meta-analysis. J Infect. (2024) 88:77–88. doi: 10.1016/j.jinf.2023.12.004 38101521

[7] Sugiyama A Takafuta T Sato T Kitahara Y Yoshinaga Y. Natural course of post-COVID symptoms in adults and children. Sci Rep. (2024) 14:3884. doi: 10.1038/s41598-024-54397-y 38365846 PMC10873293

[8] O’Hare AM Vig EK Iwashyna TJ Fox A Taylor JS Viglianti EM. Complexity and challenges of the clinical diagnosis and Management of Long COVID. JAMA Netw Open. (2022) 5:e2240332. doi: 10.1001/jamanetworkopen.2022.40332, PMID: 36326761 PMC9634500

[9] Muley A Mitra S Bhaliya B Soni S Joshi A. A systematic review and Meta-analysis to identify risk factors for developing long COVID-19 (2024) 72:68–74. doi: 10.59556/japi.72.0528 38881113

[10] Notarte KI De Oliveira MHS Peligro PJ Velasco JV Macaranas I Ver AT. Age, sex and previous comorbidities as risk factors not associated with SARS-CoV-2 infection for long COVID-19: a systematic review and Meta-analysis. J Clin Med. (2022) 11:7314. doi: 10.3390/jcm11247314, PMID: 36555931 PMC9787827

[11] Tsampasian V Elghazaly H Chattopadhyay R Debski M Naing TKP Garg P. Risk factors associated with post−COVID-19 condition: a systematic review and Meta-analysis. JAMA Intern Med. (2023) 183:566. doi: 10.1001/jamainternmed.2023.0750, PMID: 36951832 PMC10037203

[12] Van Kessel SAM Olde Hartman TC Lucassen PLBJ Van Jaarsveld CHM. Post-acute and long-COVID-19 symptoms in patients with mild diseases: a systematic review. Fam Pract. (2022) 39:159–67. doi: 10.1093/fampra/cmab076, PMID: 34268556 PMC8414057

[13] Gensichen J Zöllinger I Gagyor I Hausen A Hölscher M Janke C. Impact of the COVID-19 pandemic on people in need of care or support: protocol for a SARS-CoV-2 registry. BMJ Open. (2023) 13:e071134. doi: 10.1136/bmjopen-2022-071134, PMID: 37192790 PMC10192580

[14] Herdman M Gudex C Lloyd A Mf J Kind P Parkin D. Development and preliminary testing of the new five-level version of EQ-5D (EQ-5D-5L). Qual Life Res. (2011) 20:1727–36. doi: 10.1007/s11136-011-9903-x, PMID: 21479777 PMC3220807

[15] Mahoney FI Barthel DW. Functional evaluation: the Barthel index: a simple index of independence useful in scoring improvement in the rehabilitation of the chronically ill. Md State Med J. (1965) 14:61–5. PMID: 14258950

[16] Löwe B Spitzer RL Zipfel S Herzog W. PHQ-D: Gesundheitsfragebogen für Patienten. Pfizer GmbH: Manual Komplettversion und Kurzform (2002).

[17] Callahan CM Unverzagt FW Hui SL Perkins AJ Hendrie HC. Six-Item screener to identify cognitive impairment among potential subjects for clinical research. Med Care. (2002) 40:771–81. doi: 10.1097/00005650-200209000-00007, PMID: 12218768

[18] Krupp S Seebens A Kasper J Willkomm M Balck F. Validierung der deutschen Fassung des Six-Item Screeners: Kognitiver Kurztest mit breiten Anwendungsmöglichkeiten. Z Für Gerontol Geriatr. (2018) 51:275–81. doi: 10.1007/s00391-016-1177-z, PMID: 28093627

[19] Nasreddine ZS Phillips NA Bédirian V Charbonneau S Whitehead V Collin I. The Montreal cognitive assessment, MoCA: a brief screening tool for mild cognitive impairment. J Am Geriatr Soc. (2005) 53:695–9. doi: 10.1111/j.1532-5415.2005.53221.x, PMID: 15817019

[20] Löwe B Müller S Brähler E Kroenke K Albani C Decker O. Validierung und Normierung eines kurzen Selbstratinginstrumentes zur Generalisierten Angst (GAD-7) in einer repräsentativen Stichprobe der deutschen Allgemeinbevölkerung. PPmP - Psychother · Psychosom. Media Psychol. (2007) 57:s-2007-970669.

[21] Van Buuren S. Multiple imputation of discrete and continuous data by fully conditional specification. Stat Methods Med Res Juni. (2007) 16:219–42. doi: 10.1177/0962280206074463, PMID: 17621469

[22] Van Buuren S Boshuizen HC Knook DL. Multiple imputation of missing blood pressure covariates in survival analysis. Stat Med. (1999) 18:681–94. doi: 10.1002/(SICI)1097-0258(19990330)18:6<681::AID-SIM71>3.0.CO;2-R, PMID: 10204197

[23] Graham JW Olchowski AE Gilreath TD. How many imputations are really needed? Some practical clarifications of multiple imputation theory. Prev Sci. (2007) 8:206–13. doi: 10.1007/s11121-007-0070-9, PMID: 17549635

[24] Robert Koch Institut. (2021). SARS-CoV-2 Varianten in Deutschland. Available online at: https://public.data.rki.de/t/public/views/IGS_Dashboard/DashboardVOC?%3Aembed=y&%3AisGuestRedirectFromVizportal=y (Accessed December 11, 2024).

